# Evaluating the clinical trends and benefits of low‐dose computed tomography in lung cancer patients

**DOI:** 10.1002/cam4.4229

**Published:** 2021-09-16

**Authors:** Edmund M. Qiao, Rohith S. Voora, Vinit Nalawade, Nikhil V. Kotha, Alexander S. Qian, Tyler J. Nelson, Michael Durkin, Lucas K. Vitzthum, James D. Murphy, Tyler F. Stewart, Brent S. Rose

**Affiliations:** ^1^ Department of Radiation Medicine and Applied Sciences University of California San Diego La Jolla California USA; ^2^ Veterans Health Administration San Diego Health Care System La Jolla California USA; ^3^ Department of Radiation Oncology Stanford University Stanford California USA; ^4^ Division of Hematology‐Oncology Department of Internal Medicine University of California San Diego La Jolla California USA

**Keywords:** cancer screening, CT screening, LDCT, low‐dose CT, lung cancer

## Abstract

**Background:**

Despite guideline recommendations, utilization of low‐dose computed tomography (LDCT) for lung cancer screening remains low. The driving factors behind these low rates and the real‐world effect of LDCT utilization on lung cancer outcomes remain limited.

**Methods:**

We identified patients diagnosed with non‐small cell lung cancer (NSCLC) from 2015 to 2017 within the Veterans Health Administration. Multivariable logistic regression assessed the influence of LDCT screening on stage at diagnosis. Lead time correction using published LDCT lead times was performed. Cancer‐specific mortality (CSM) was evaluated using Fine–Gray regression with non‐cancer death as a competing risk. A lasso machine learning model identified important predictors for receiving LDCT screening.

**Results:**

Among 4664 patients, mean age was 67.8 with 58‐month median follow‐up, 95% CI = [7–71], and 118 patients received ≥1 screening LDCT before NSCLC diagnosis. From 2015 to 2017, LDCT screening increased (0.1%–6.6%, mean = 1.3%). Compared with no screening, patients with ≥1 LDCT were more than twice as likely to present with stage I disease at diagnosis (odds ratio [OR] 2.16 [95% CI 1.46–3.20]) and less than half as likely to present with stage IV (OR 0.38 [CI 0.21–0.70]). Screened patients had lower risk of CSM even after adjusting for LDCT lead time (subdistribution hazard ratio 0.60 [CI 0.42–0.85]). The machine learning model achieved an area under curve of 0.87 and identified diagnosis year and region as the most important predictors for receiving LDCT. White, non‐Hispanic patients were more likely to receive LDCT screening, whereas minority, older, female, and unemployed patients were less likely.

**Conclusions:**

Utilization of LDCT screening is increasing, although remains low. Consistent with randomized data, LDCT‐screened patients were diagnosed at earlier stages and had lower CSM. LDCT availability appeared to be the main predictor of utilization. Providing access to more patients, including those in diverse racial and socioeconomic groups, should be a priority.

## Graphical abstract

In this retrospective cohort study from 2015 to 2017, we found that low‐dose computed tomography (LDCT) screening usage remains low (1.3%), significantly increases the odds of stage I diagnosis (odds ratio [OR] 2.16), decreases odds of stage IV diagnosis (OR 0.38), and decreases risk of cancer‐specific mortality (subdistribution hazard ratio 0.60). LDCT usage appears to be driven by regional and temporal differences LDCT access. Increasing access for all patients should be a priority toward reducing lung cancer mortality.

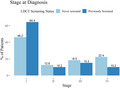

## 1 INTRODUCTION

Lung cancer remains the leading cause of cancer mortality within the United States. Most cases of lung cancer are detected at later stages, and 5‐year survival is 21%.^1^ Current data support the benefit of low‐dose computed tomography (LDCT) screening on lung cancer mortality for patients diagnosed with non‐small cell lung cancer (NSCLC).^2^ Recent guideline recommendations from the United States Preventive Services Task Force (USPSTF) have endorsed LDCT screening high‐risk patients.^3^ Despite these recommendations, national LDCT screening remains low. Single‐year studies have estimated LDCT utilization ranging from around 2% to 4% of eligible patients per year.^4^, ^5^, ^6^

The driving forces behind underutilization of LDCT screening are not well understood. As a relatively new guideline, physicians may be unfamiliar with proper screening criteria or feel uncertain about the strength of existing research in favor of LDCT screening. Patients may be less inclined to opt into yearly screenings that until recently were not recommended. To aid physicians and patients’ informed decision‐making regarding LDCT screening, additional research is needed to more thoroughly characterize its potential benefit, especially following the establishment and recent expansion of national screening guidelines.^7^ Furthermore, understanding the current trends surrounding LDCT screening may identify factors contributing to the low prevalence of national LDCT screening.

Using the Veterans Affairs Informatics and Computing Infrastructure (VINCI) database, we conducted a large retrospective analysis to investigate the clinical outcomes of NSCLC patients eligible for LDCT screening and identify predictors for receiving LDCT screening. Regression analysis estimated the impact of LDCT screening on stage at diagnosis. Multivariable competing risk survival analysis evaluated the impact of LDCT screening on lead time adjusted cancer‐specific mortality (CSM). We employed a machine learning approach to identify the most important predictors for receiving LDCT screening in eligible patients.

## 2 METHODS

### 2.1 Data source

Veterans Affairs Informatics and Computing Infrastructure contains patient‐level electronic health record information for all veterans within the Veterans Affairs (VA) healthcare system. The data include demographic information, clinical notes, imaging, operative, and pathology reports. VINCI includes tumor registry information gathered by registrars at individual VA sites, with data recorded according to standard protocols.^8^, ^9^

### 2.2 Study population

In accordance with USPSTF recommendations for the study period, the study population was restricted to patients aged 55–80 with previously documented nicotine dependence. Diagnostic and procedural codes indicated nicotine dependence and LDCT screening^5^ (Appendix 1). We identified patients diagnosed with NSCLC from 2015 to 2017, which reflect when LDCT screening coding and VINCI diagnosis dates became available. Patients missing clinical staging, survival follow‐up, cause of death, or primary payer other than the VA were excluded (Figure S1). The following covariables were included: LDCT screening, primary care provider (PCP) visits, age at diagnosis, race, ethnicity, year of diagnosis, marital status, employment status, education, VA site, Charlson comorbidity scores, and median income by zip code. TNM staging adhered to American Joint Committee on Cancer, 7th edition. Population statistics for VA sites were matched with data from the United States Census Bureau.

Individual LDCT screening rate was calculated as follows:

$$\frac{\sum_{i=0}^{n}x_{i}}{\mathrm{EligibleYears}},$$

where *n* is the number of years where a patient received ≥ 1 LDCT.

*EligibleYears* is the set of years where the patient met clinical criteria for receiving a LDCT screening test.

To estimate healthcare utilization, PCP visit rate was calculated as above, but utilized the 5 years prior to date of diagnosis as *EligibleYears*. Procedural codes indicating a primary or preventative visit were used for PCP visits.

### 2.3 Regression analysis

Patient demographic, clinical, and treatment characteristics are reported in Table 1. Stage at diagnosis and CSM were primary endpoints, detailed in the Appendix 1. The cause of death indicating death from any cancer was grouped into our event, and the cause of death from any other means grouped into the non‐cancer death competing event.

**TABLE 1:** Patient characteristics and demographics. Patients further stratified by 0 previous low‐dose CT (LDCT) screens versus ≥1 LDCT (“Never‐Screened” vs. “LDCT Screened”). TNM staging adhered to American Joint Committee on Cancer, 7th edition. This period included patients diagnosed with NSCLC from 2015 to 2017 with a median follow‐up of 58 months (95% confidence interval = [7 months‐71 months]) with a final follow‐up date cutoff of 1 February 2021. *p*‐values calculated with chi‐square for multi‐group categorical variables, two‐proportion *z*‐test for single proportion variables, and student's *t*‐test for continuous variables. Continuous variables presented as mean (SD), categorical as total (%)

Variable	Total	Never‐screened	LDCT screened	*p*‐value
Number	4664	4546	118	
LDCT screening rate (%)	1.3 (8.0)	0 (0)	49.6 (11.2)	<0.01
Age	67.8 (5.6)	67.8 (5.6)	67.3 (4.9)	0.23
Race				0.12
White	3819 (81.9%)	3714 (81.7%)	105 (89.0%)	
Black	745 (16.0%)	733 (16.1%)	12 (10.2%)	
Other	100 (2.1%)	99 (2.2%)	1 (0.8%)	
Ethnicity				0.47
Hispanic	43 (0.9%)	43 (0.9%)	0 (0.0%)	
Non‐Hispanic	4606 (98.8%)	4488 (98.8%)	118 (100%)	
Unknown	15 (0.3%)	15 (0.3%)	0 (0.0%)	
Year diagnosed				<0.01
2015	2424 (52.0%)	2422 (53.3%)	2 (1.7%)	
2016	2101 (45.1%)	2004 (44.1%)	99 (83.9%)	
2017	139 (2.9%)	120 (2.6%)	17 (14.4%)	
Clinical T stage				0.03
T1	2523 (54.1%)	2444 (53.8%)	79 (66.9%)	
T2	1054 (22.6%)	1032 (22.7%)	22 (18.6%)	
T3	551 (11.8%)	542 (11.9%)	9 (7.6%)	
T4	536 (11.5%)	528 (11.6%)	8 (6.9%)	
Clinical N stage				0.75
N0	3051 (65.4%)	2959 (65.1%)	92 (78.0%)	
N1	337 (7.2%)	330 (7.3%)	7 (5.9%)	
N2	868 (18.7%)	855 (18.8%)	13 (11.0%)	
N3	408 (8.7%)	402 (8.8%)	6 (5.1%)	
Clinical M stage				<0.01
M0	3639 (77.9%)	3533 (77.7%)	106 (89.7%)	
M1	1025 (22.1%)	1013 (22.3%)	12 (10.3%)	
Histology				0.78
Adenocarcinoma	2039 (43.7%)	1987 (43.7%)	52 (44.1%)	
Squamous cell	1697 (36.4%)	1657 (36.4%)	40 (33.9%)	
Other/not listed NSCLC	928 (19.9%)	902 (19.9%)	26 (22.0%)	
Mean income^a^ (10k)	50.0 (17.5)	49.8 (17.4)	55.1 (19.9)	<0.01
Mean bachelors^a^ (%)	15.2 (7.4)	15.2 (7.4)	17.0 (8.2)	0.02
PCP visit rate (%)	92.5 (17.6)	92.5 (17.5)	89.8 (0.22)	0.18

_Abbreviations: NSCLC, non‐small cell lung cancer; PCP, primary care provider_.

^a^ _By zip code_.

**FIGURE 1:**
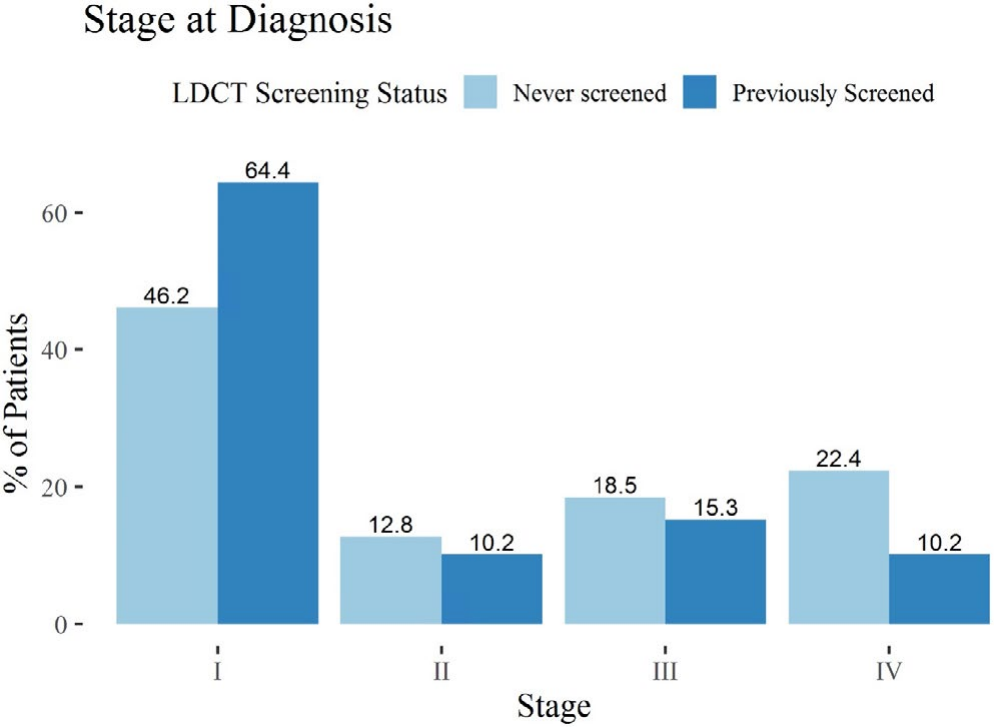
Stage at diagnosis stratified by low‐dose computed tomography (LDCT) screening status. Patients stratified by 0 previous low‐dose CT screens versus ≥1 LDCT (“Never Screened” vs. “Previously Screened”)

**FIGURE 2:**
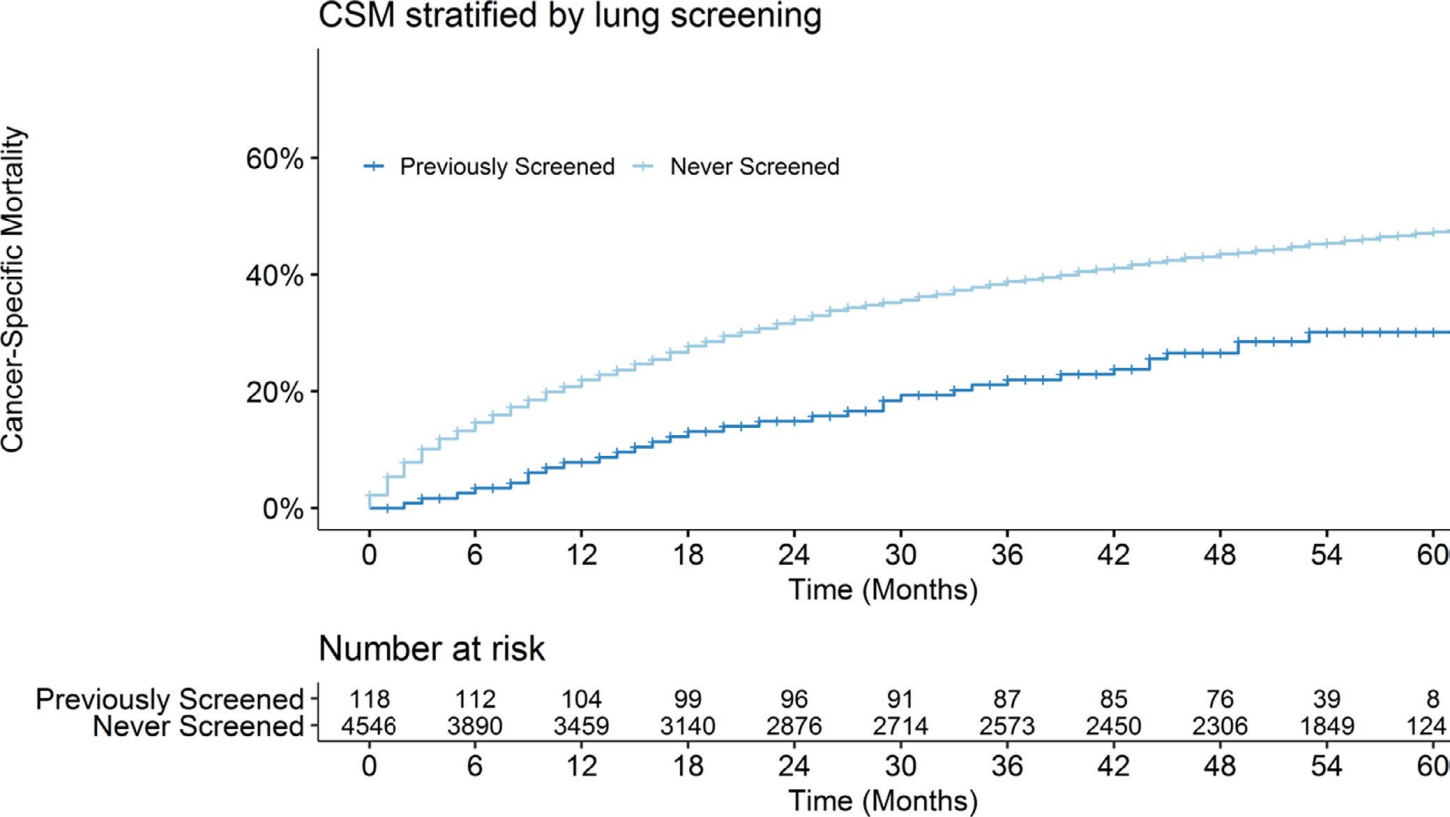
Cumulative incidence of lung CSM. Patients stratified by no previous low‐dose computed tomography (LDCT) screens versus ≥1 LDCT (“Never Screened” vs. “Previously Screened”). CSM, cancer‐specific mortality

We fit separate multivariable logistic regression models assessing the impact of LDCT screening on stage I and stage IV at diagnosis while adjusting for the aforementioned covariates (Table S1). For CSM, we fit multivariable Fine–Gray competing risk regression with non‐cancer death as a competing risk and CSM as the endpoint. The mean lead time afforded to LDCT screen‐detected lung cancer has been previously estimated.^10^ We corrected our survival times using methods described by Duffy et al and the average estimated lead times from the National Lung Screening Trial and Dutch–Belgian lung cancer screening trial^11^, ^12^(Table S2). We fit competing risk regression with these corrected survival times to mitigate survival benefit attributed to lead time (Table S1). The final date cutoff for survival analysis was 1 February 2021. Similar to studies utilizing lead time adjusted survival analysis,^13^, ^14^, ^15^ we excluded cancer stage in our adjusted survival regression. Including both staging along with lead time correction would likely obscure the effect of screening on CSM by overcorrecting for benefits from stage shift. In addition, increasing evidence suggests that LDCT may preferentially detect lepidic pattern adenocarcinomas that represent a lower risk subtype,^16^ and sensitivity analysis excluding adenocarcinomas was performed. For each cohort, patients were stratified into “previously screened” if they received ≥1 screening LDCT within the study period and “never‐screened” if they never received a screening LDCT. The proportions of stage at diagnosis are shown in Figure 1, and difference of proportions was evaluated with two‐sided z‐tests. Cumulative incidence functions were fit for these models (Figure 2). Log‐rank test evaluated differences in survival curves.

### 2.4 Identifying predictors for LDCT screening

A lasso model identified important predictors for receiving LDCT screening. Events were categorized as “previously screened” versus “never‐screened” with the aforementioned criteria. To evaluate model performance, the data were split 75%/25% into training/testing data balanced by our event. Training models were evaluated with bootstrapping methods. We hypothesized that availability of LDCT services may be influenced by VA site and included de‐identified VA site variable along with previously described covariates. The highest performing training model was selected by area under the curve (AUC), with an AUC of 1.0 indicating perfect prediction. Important features were identified by relative contribution of their scaled coefficients. This model predicted receipt of LDCT for patients in our 25% testing data. Sensitivity analysis without initial cohort exclusion criteria was performed to capture patients eligible for screening but lacked covariates necessary for the previous regression analysis (Figure S2). Statistical tests were two‐sided, with *p* < 0.05 considered significant. Analyses were conducted with R, v3.5.1 and survival (v3.2), ggplot2 (v3.3.3), and tidymodels (v0.1.2) packages.

## 3 RESULTS

### 3.1 Baseline characteristics

The cohort included 4664 patients diagnosed with lung cancer and previously documented nicotine dependence. Among these, 118 patients received ≥1 LDCT screen prior to diagnosis and 4546 patients had not. Average age at diagnosis was 67.8 with median follow‐up of 58 months, 95% confidence interval (CI) = [7–71 months]. In total, 43.7% of cases were adenocarcinoma, 36.4% of cases were squamous cell, and 19.9% of cases were other NSCLC that included cases lacking additional histology data. Table 1 summarizes patient demographic and tumor characteristics. Average overall LDCT screening rate for VA was 1.3%, and from 2015 to 2017 the LDCT screening rate rose from 0.1% to 6.6%. There was negligible correlation between PCP visit rate and LDCT screening rate (*r* = −0.02, *p* < 0.01). In general, patients who received LDCT screening were more likely have higher education, higher median income, and be diagnosed in later years (Table 1).

### 3.2 Disease stage at diagnosis

Screened patients were more likely to be diagnosed with stage I disease (64.4% vs. 46.2%, *p* < 0.01) and less likely to be diagnosed with stage IV disease (10.2% vs. 22.4%, *p* < 0.01), Figure 1. On multivariable logistic regression, screened patients were more than twice as likely to present with stage I disease at diagnosis (OR = 2.16, 95% CI = [1.46–3.20], *p* < 0.01) and less than half as likely to be diagnosed with metastatic disease (OR = 0.38, 95% CI = [0.21–0.70], *p* < 0.01), Table S1. In addition, higher PCP visit rate was also significantly associated with higher odds of stage I disease at diagnosis (OR = 1.13, 95% CI = [1.05–1.23], *p* < 0.01) and lower odds of stage IV disease at diagnosis (OR = 0.84, 95% CI = [0.77–0.92], *p* < 0.01). Additional covariates significantly associated with stage at diagnosis were Charlson scores ≥2, tumor histology subtype, and age. Sensitivity analysis restricting PCP visits to the same *EligibleYears* as LDCT screening did not impact conclusions.

### 3.3 Cancer‐specific mortality

We found a significant difference between the cumulative incidence functions of CSM between previously screened and never‐screened patients (*p* < 0.01). Previously screened patients had significantly reduced cumulative incidence of CSM at median follow‐up of 58 months (30.3% vs. 46.6%, *p* < 0.01), Figure 2. On multivariable regression, screened patients had 40% reduced risk of CSM even after adjusting for LDCT lead time (subdistribution hazard ratio [SHR] = 0.60, 95% CI = [0.42–0.85], *p* < 0.01). Other factors significantly associated with increased CSM were higher Charlson scores and single marital status. The results of the full multivariable regression model are summarized in Table S1. Sensitivity analyses using minimum and maximum estimated lead times and analysis excluding adenocarcinomas did not impact our results (data not shown).

### 3.4 Predictors for receiving LDCT screening

**FIGURE 3:**
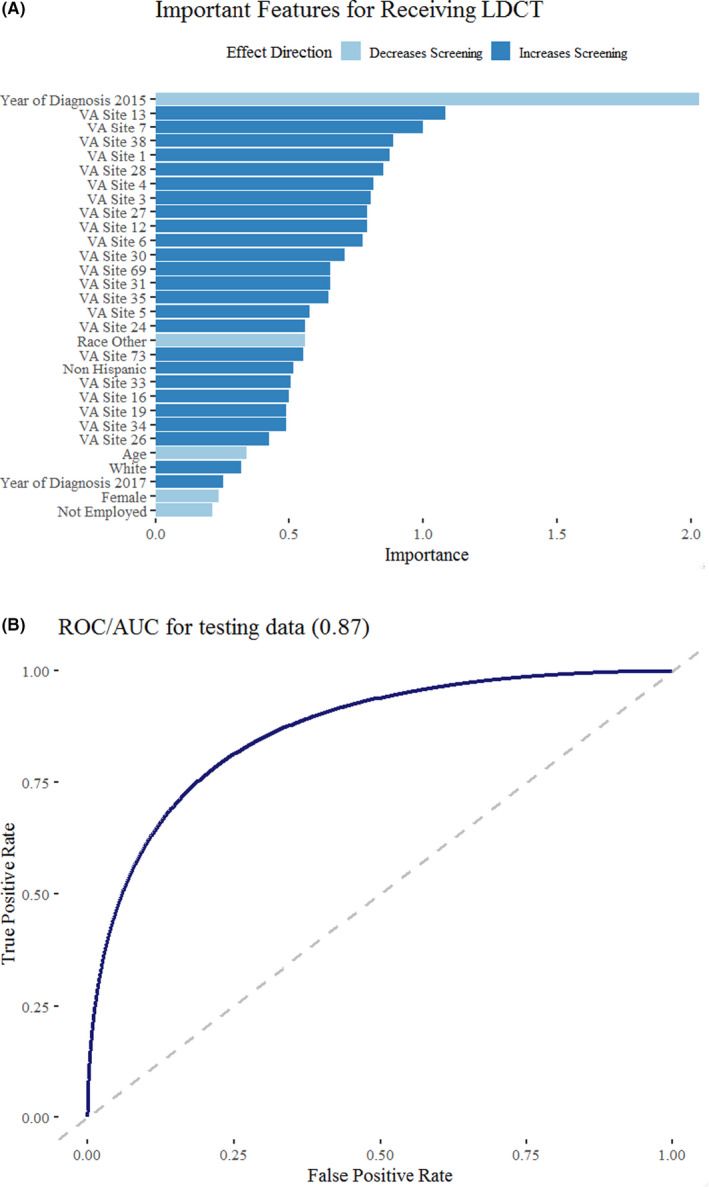
Lasso model for identifying important predictors for low‐dose computed tomography (LDCT) screening. Panel (A) shows the top 30 predictors identified by the lasso model. Importance was calculated as the normalized weight for each coefficient of the lasso model. Panel (B) shows the receiver operator curve area under the curve (ROC/AUC) for 25% testing dataset

The lasso model achieved an AUC of 0.87 for predicting receipt of LDCT on our testing dataset. The most important predictors identified from our lasso model were year of diagnosis and specific VA site. Our model suggests that patients diagnosed in 2015 were less likely to receive LDCT screening while patients diagnosed in 2017 were more likely. Distinct VA sites were also identified as important predictors that increased the likelihood of LDCT screening. The model also suggests that non‐Hispanic, White patients were more likely to receive screening, while minorities, older, female, and unemployed patients were less likely. These findings are summarized in Figure 3. In general, VA sites identified as more likely to perform LDCT screening served larger regional populations than those identified as less likely. The median population of higher screening VA sites was 296,943, compared to 157,206 in the lower screening VA sites. Table S3 summarizes the number of total patients and proportion of LDCT‐screened patients stratified by VA site. There was a moderate correlation (Pearson's *r* = 0.6, *p* < 0.01) between the total number of patients per VA site and proportion receiving LDCT screening.

## 4 DISCUSSION

Randomized trials have shown mortality benefit of LDCT screening for high‐risk populations,^2^, ^17^ and in early 2021, the USPSTF expanded eligibility for LDCT screening to encompass additional patients.^7^ However, nationwide uptake of LDCT screening remains poor.^18^ Findings from this study indicate that LDCT utilization within the VA is low, though increasing year‐by‐year. Additionally, we found LDCT screening is associated with improved lung cancer outcomes. Patients who underwent ≥1 LDCT screen prior to their diagnosis were more likely to present with stage I disease, less likely to present with stage IV disease, and had improved lead time adjusted CSM. LDCT utilization appears most influenced by year of diagnosis and VA site. However, patient characteristics including non‐Hispanic ethnicity and White race appear to increase the likelihood of LDCT screening. Improving access to care for all patients, including those in diverse racial and socioeconomic groups, should be a priority to reduce lung cancer mortality.

We found that between 2015 and 2017, 1.3% of eligible VA patients received LDCT screening, lower than previously reported. In a Medicare cohort, Tailor et al report a 4.1% utilization rate, though their analysis was restricted to 2016. By limiting our cohort to 2016, we find a comparable LDCT rate of 4.7%, though Nishi et al describe a LDCT utilization of 2.2% in a similar Medicare cohort. Huo et al describe a screening rate of 3.8% for 2015, compared to ours of 0.1%. The variance in LDCT utilization between our and previous studies could be attributed to differences in assigning smoking eligibility criteria and availability of LDCT screening‐specific procedural codes. We relied on previously described methods using procedural and diagnostic codes^5^ while other studies matched smoking data from US Census records or self‐reported surveys. Few studies have evaluated the uptake of nationwide LDCT screening, and even fewer have looked at more consecutive years since the USPSTF first implemented their screening guidelines. We find rates and trends comparable with national estimates and previous studies. Furthermore, our results reveal a promising upward trend of LDCT screening by year from 0.1% to 6.6%. This could indicate that providers are increasingly adhering to LDCT screening guidelines.

The real‐world clinical benefit of LDCT screening is an understudied area of research. Our results are consistent with existing randomized control trials.^2^, ^17^ We found that LDCT screening was significantly associated with greater than twice the odds of stage I disease at diagnosis (OR=2.16, 95% CI = [1.46–3.20], *p* < 0.01) and less than half the odds of stage IV disease at diagnosis (OR = 0.38, 95% CI = [0.21–0.70], *p* < 0.01). Currently, the majority of lung cancer cases are diagnosed at late stages.^1^ Earlier diagnosis of lung cancer enables definitive treatment measures crucial for survival. The 5‐year survival of localized disease is 59%, compared to 6% for distant disease.^1^ In our multivariable survival analysis, LDCT screening was associated with 40% decreased risk of CSM (SHR = 0.60, 95% CI = [0.42–0.85], *p* < 0.01). By accounting for lead time in our survival analysis, this reduction more accurately represents the benefit of increased access to curative treatment. Overall, these results suggest that real‐world implementation of LDCT screening may be improving lung cancer outcomes for high‐risk patients.

Our analysis used a machine learning approach to identify predictors that may influence utilization of LDCT screening. To this end, our lasso model achieved a strong testing AUC of 0.87, lending credence to its predictive accuracy and appropriate selection of predictive features. We found predictors potentially reflecting access to care or radiologic services––year of diagnosis, VA site of care––as the most important predictors for receiving LDCT screening. The increasing LDCT usage with increasing year of diagnosis likely reflects increasing rollout of LDCT recommendations among healthcare professionals. Interestingly, we found that PCP visit rate was negligibly correlated with LDCT screening rate (*r* = −0.02, *p* < 0.01). PCPs are likely properly screening high‐risk patients, and studies have indicated the majority of LDCT orders for high‐risk patients originate from PCPs.^19^ Therefore, we believe our findings suggest that the driving force behind low LDCT utilization could be attributed to regional differences in availability of radiologic services rather than overall access to healthcare. The benefit of increased healthcare utilization on proper LDCT screening is likely blunted by the lack of available screening facilities. CT utilization has been shown to cluster regionally,^6^ with rural regions less likely to provide imaging services.^20^ Smokers in rural areas are already known to be an at‐risk group for poor LDCT screening.^21^ Our results reflect these regional differences, as the median population of VA stations providing higher LDCT rates was about 2× the median population of those that had lower LDCT rates. Addressing these regional gaps and overall organizational readiness for LDCT implementation remains an important area of research as screening recommendations continue to expand.^22^

Overall, our machine learning model ranked patient demographics as less important than predictors indicative of access to care, yet the model still highlighted differences between racial and socioeconomic factors. White, non‐Hispanic patients were identified as more likely to receive LDCT screening, while minorities, older, female, and unemployed patients were less likely. Racial and socioeconomic disparities for other cancer screening are well documented, with minorities and lower socioeconomic status patients less likely to receive guideline screening or treatment.^23^ Our analysis identifies these patients as potential at‐risk groups for poor LDCT screening as well. As future expansion of radiologic services will likely reduce the impact regional differences on LDCT screening, these racial and socioeconomic disparities will likely persist unless increased, targeted efforts are undertaken for these at‐risk patients.

This study has limitations worth nothing. First, we relied on diagnostic and procedural coding to identify nicotine dependence and identify LDCT screening cases. Misclassification of these variables may introduce bias into our analysis. This misclassification seemed to decrease in subsequent years, and we believe this misclassification would be more likely to attenuate than heighten our regression results by reducing the effect size conveyed by LDCT screening. Additional studies are needed to address this limitation. Second, our survival analysis is limited by lead time bias. Increased lead time itself is not inherently a negative factor, as one goal of cancer screening is earlier detection for increased chances of curative treatment. Lead time bias can occur when screen‐detected cancers represent a more indolent subtype. We attempted to control for lead time bias with previously established methods to mitigate this effect^11^ and performed sensitivity analysis removing more indolent lepidic adenocarcinomas from our analysis. LDCT screening has only recently been established, and available data are limited by proper usage of new codes and database curation of these variables. We expect our analysis could be refined with additional study period years and available LDCT‐specific coding variables. Additional studies with longer follow‐up periods are needed to further characterize the impact of these relevant variables on our endpoints.

## 5 CONCLUSIONS

Despite expanded USPSTF recommendations and multiple large randomized trials demonstrating a significant reduction in CSM, LDCT screening remains low both nationwide and within the VA health system. Patients screened with LDCT were diagnosed at earlier stages and less likely to die from their cancer. Our findings are consistent with previous randomized data and support increased utilization of LDCT screening. The driving forces behind low screening utilization appear to reflect decreased access to LDCT services. Improving access to care for patients, including those in diverse racial and socioeconomic groups, should be a priority to reduce lung cancer mortality.

## CONFLICT OF INTEREST

The authors report no conflict of interest.

## AUTHOR CONTRIBUTIONS

Conceptualization, data curation, formal analysis, investigation, methodology, software, validation, and visualization: Edmund M. Qiao, Rohith S. Voora, Vinit Nalawade, Brent S. Rose, and Tyler F. Stewart; Project administration, resources, and supervision: Brent S. Rose and Tyler F. Stewart; Writing––original draft: Edmund M. Qiao, Rohith S. Voora, Brent S. Rose, and Tyler F. Stewart; Writing––review and editing: Edmund M. Qiao, Nikhil V. Kotha, Alexander S. Qian, Tyler J. Nelson, Michael Durkin, Lucas K. Vitzthum, James D. Murphy, Tyler F. Stewart, and Brent S. Rose.

## ETHICS STATEMENT/IRB APPROVAL

150169 on 10/17/2019, expires 11/09/2021.

## Supporting information

Fig S1

Fig S2

Table S1

Table S2

Table S3

## Data Availability

The data that support the findings of this study are available from Veterans' Health Administration. Restrictions apply to the availability of these data, which were used under license for this study. Data are available for request at https://www.hsrd.research.va.gov/for_researchers/vinci/ with the permission of the Veterans' Health Administration.

## APPENDIX 1. Information on variable and endpoint selection

- Diagnosis and procedural codes used for cohort and covariate selection.
- For nicotine documentation, International Classification of Diseases, Ninth Revision codes “305.1” and “V15.82” and International Classification of Diseases, Tenth Revision codes “F17.21” and “Z87.891” were used.
- For low‐dose CT screening, Healthcare Common Procedural Coding System and Current Procedural Terminology codes “G0297,” “S8032,” and “71271” were used.
- For primary care utilization, Current Procedural Terminology codes “99201,” “99202,” “99203,” “99204,” “99205,” “99211,” “99212,” “99213,” “99214,” “99215,” “99381,” “99382,” “99383,” “99384,” “99385,” “99386,” “99387,” “99391,” “99392,” “99393,” “99394,” “99395,” “99396,” “99397,” “99401,” “99402,” “99403,” “99404,” “99408,” and “99409” were used.
- Diagnosis and procedural codes used for cohort and covariate selection.
- Stage at diagnosis was evaluated using staging variables from the Veterans Affairs (VA) cancer registry.
- Cancer‐specific mortality was evaluated using the VA cancer registry cross‐referenced with National Death Index data and VA clinical notes.
