# Supplementary figures and images for: Evaluating the clinical trends and benefits of low‐dose computed tomography in lung cancer patients
Source: Cancer Med. 2021 Sep 16;10(20):7289–97. doi: 10.1002/cam4.4229 (PMC8525167; doi:10.1002/cam4.4229)

**Supplementary figure 1. Selection criteria.**

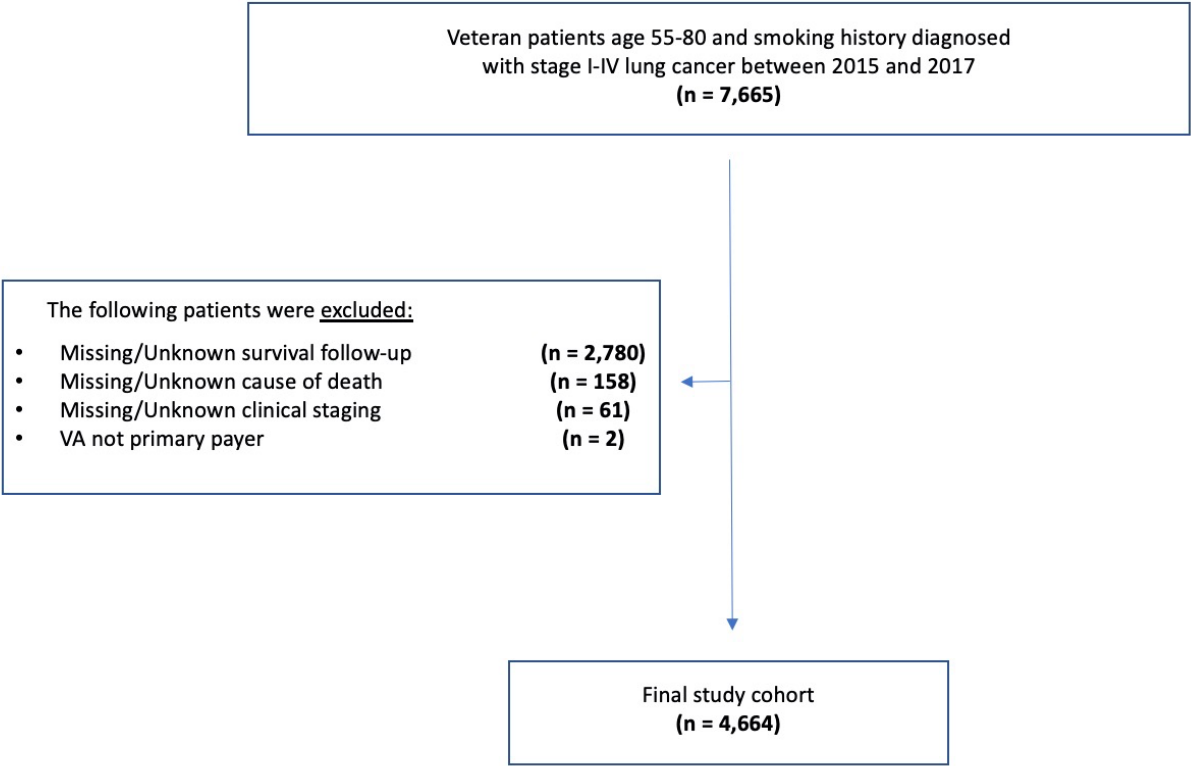

Supplement: Supplementary file 1 — Fig S1 [file CAM4-10-7289-s001.pdf]
