# Supplementary material for: Evaluating the clinical trends and benefits of low‐dose computed tomography in lung cancer patients
Source: Cancer Med. 2021 Sep 16;10(20):7289–97. doi: 10.1002/cam4.4229 (PMC8525167; doi:10.1002/cam4.4229)

Supplemental Figure 2. Lasso regression with no excluded patients. Panel A shows the top 30 predictors identified by the lasso model. Importance was calculated as the normalized weight for each coefficient of the lasso model. Panel B shows displays the receiver operator curve area under the curve (ROC/AUC) for 25% testing dataset.

A)

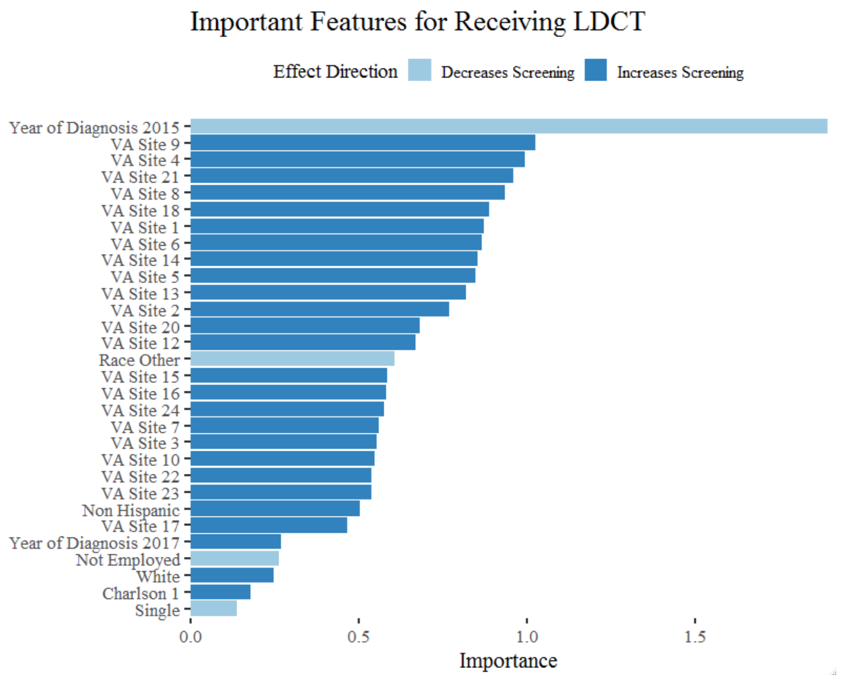

B)

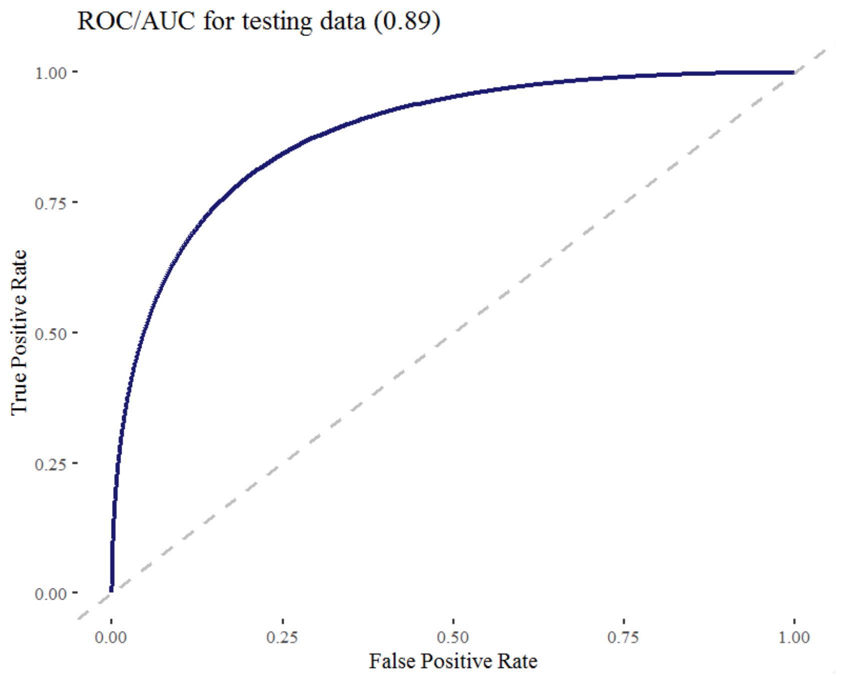

Supplement: Supplementary file 2 — Fig S2 [file CAM4-10-7289-s003.pdf]
