# Supplementary material for: Evaluating the clinical trends and benefits of low‐dose computed tomography in lung cancer patients
Source: Cancer Med. 2021 Sep 16;10(20):7289–97. doi: 10.1002/cam4.4229 (PMC8525167; doi:10.1002/cam4.4229)
Supplement: Supplementary file 3 — Table S1 [file CAM4-10-7289-s004.docx]

**Supplemental Table 1. Full model outputs for regression models.** Logistic regression performed with stage I or stage IV disease at diagnosis as the endpoint. Fine-Gray competing risk regression performed with cancer mortality as the endpoint and non-cancer death as competing event, survival adjusted for lead time.

| **Characteristic** | **Endpoint**  OR/SHR [95%CI] | | |
| --- | --- | --- | --- |
|  | **Stage I at Diagnosis** | **Stage IV at Diagnosis** | **Cancer-Specific Mortality** |
| LDCT screened | 2.16 [1.46-3.20] | 0.38 [0.21-0.70] | 0.60 [0.42-0.85] |
| Age | 1.01 [1.002-1.02] | 1.00 [0.99-1.01] | 1.00 [1.00-1.01] |
| Sex |  |  |  |
| Male | 0.77 [0.55-1.08] | 1.05 [0.70-1.57] | 1.24 [0.96-1.61] |
| Female | 1 | 1 | 1 |
| Race |  |  |  |
| White | 1 | 1 | 1 |
| Black | 0.88 [0.75-1.04] | 1.17 [0.97-1.42] | 1.00 [0.89-1.12] |
| Other | 0.77 [0.51-1.15] | 1.22 [0.77-1.96] | 1.26 [0.99-1.59] |
| Ethnicity |  |  |  |
| Non-Hispanic | 1 | 1 | 1 |
| Hispanic | 0.72 [0.39-1.32] | 0.85 [0.39-1.86] | 0.86 [0.55-1.36] |
| Unknown | 0.75 [0.26-2.15] | 0.26 [0.03-2.00] | 0.64 [0.28-1.47] |
| Year of Diagnosis |  |  |  |
| 2015 | 1 | 1 | 1 |
| 2016 | 0.94 [0.83-1.06] | 1.00 [0.87-1.16] | 0.91 [0.48-1.08] |
| 2017 | 0.94 [0.66-1.34] | 0.96 [0.62-1.49] | 0.79 [0.19-1.34] |
| Charlson Score |  |  |  |
| 0 | 1 | 1 | 1 |
| 1 | 1.08 [0.91-1.28] | 1.20 [0.97-1.47] | 1.19 [1.05-1.34] |
| 2+ | 1.16 [1.003-1.34] | 1.19 [1.01-1.42] | 1.24 [1.12-1.38] |
| Histology  Adenocarcinoma  Squamous Cell  Other NSCLC | 1  0.85 [0.75-0.97]  1.42 [1.22-1.66] | 1  0.56 [0.47-0.66]  0.87 [0.73-1.05] | 1  1.00 [0.91-1.10]  0.97 [0.86-1.09] |
| Employment |  |  |  |
| Other/Not Employed | 1 | 1 | 1 |
| Employed | 1.00 [0.86-1.17] | 1.00 [0.84-1.22] | 1.00 [0.90-1.11] |
| Marital Status |  |  |  |
| Married | 1 | 1 | 1 |
| Other/Not Married | 0.92 [0.83-1.04] | 1.11 [0.96-1.28] | 1.17 [1.07-1.28] |
| % College Education^a^ | 0.97 [0.88-1.07] | 0.97 [0.88-1.07] | 0.95 [0.90-1.00] |
| Median Income^a^ | 1.07 [0.98-1.19] | 1.05 [0.96-1.16] | 0.98 [0.93-1.05] |
| PCP visit rate | 1.13 [1.05–1.23] | 0.84 [0.77–0.92] | 0.95 [0.90–1.01] |

_LDCT = low-dose CT. PCP = primary care provider, NSCLC = non-small cell lung cancer, OR = odds ratio. SHR = subdistribution hazard ratio. CI = Confidence interval, a = by zip code._
