# Supplementary material for: Evaluating the clinical trends and benefits of low‐dose computed tomography in lung cancer patients
Source: Cancer Med. 2021 Sep 16;10(20):7289–97. doi: 10.1002/cam4.4229 (PMC8525167; doi:10.1002/cam4.4229)
Supplement: Supplementary file 4 — Table S2 [file CAM4-10-7289-s002.docx]

**Supplemental Table 2. Estimated lead-times (months) for low dose CT (LDCT) screening for lung cancer.** Adapted from Benbassat et al.

| Trial | Lead-time (estimated) | Number of Patients |
| --- | --- | --- |
| National Lung Cancer Screening  Men 55-74, moderate smoker  Patients 55-69, heavy smoker | 15.6  24.0 | 53,454 |
| Dutch–Belgian lung cancer screening trial  Men 55-74, moderate smoker  Patients 55-69, heavy smoker | 13.2  13.2 | 13,195 |
| Mean | 13.2 | 33,324 |
