# Supplementary material for: Evaluating the clinical trends and benefits of low‐dose computed tomography in lung cancer patients
Source: Cancer Med. 2021 Sep 16;10(20):7289–97. doi: 10.1002/cam4.4229 (PMC8525167; doi:10.1002/cam4.4229)
Supplement: Supplementary file 5 — Table S3 [file CAM4-10-7289-s005.docx]

**Supplemental Table 3. Patients screened by Veterans’ Affairs Center.**

| VA Site | Total Patients | Number Screened | Proportion Screened |
| --- | --- | --- | --- |
| 1 | 204 | 6 | 0.03 |
| 2 | 182 | 4 | 0.02 |
| 3 | 33 | 3 | 0.09 |
| 4 | 121 | 12 | 0.10 |
| 5 | 102 | 9 | 0.09 |
| 6 | 137 | 8 | 0.06 |
| 7 | 52 | 1 | 0.02 |
| 8 | 119 | 9 | 0.08 |
| 9 | 170 | 15 | 0.09 |
| 10 | 33 | 6 | 0.18 |
| 11 | 40 | 1 | 0.03 |
| 12 | 60 | 6 | 0.10 |
| 13 | 101 | 4 | 0.04 |
| 14 | 159 | 5 | 0.03 |
| 15 | 43 | 5 | 0.12 |
| 16 | 55 | 5 | 0.09 |
| 17 | 45 | 1 | 0.02 |
| 18 | 184 | 3 | 0.02 |
| 19 | 99 | 2 | 0.02 |
| 20 | 74 | 4 | 0.05 |
| 21 | 148 | 6 | 0.04 |
| 22 | 77 | 1 | 0.01 |
| 23 | 61 | 1 | 0.02 |
| 24 | 49 | 1 | 0.02 |
| 25 | 110 | 0 | 0 |
| 26 | 54 | 0 | 0 |
| 27 | 60 | 0 | 0 |
| 28 | 36 | 0 | 0 |
| 29 | 118 | 0 | 0 |
| 30 | 39 | 0 | 0 |
| 31 | 34 | 0 | 0 |
| 32 | 32 | 0 | 0 |
| 33 | 19 | 0 | 0 |
| 34 | 99 | 0 | 0 |
| 35 | 64 | 0 | 0 |
| 36 | 18 | 0 | 0 |
| 37 | 143 | 0 | 0 |
| 38 | 24 | 0 | 0 |
| 39 | 33 | 0 | 0 |
| 40 | 117 | 0 | 0 |
| 41 | 60 | 0 | 0 |
| 42 | 34 | 0 | 0 |
| 43 | 34 | 0 | 0 |
| 44 | 42 | 0 | 0 |
| 45 | 8 | 0 | 0 |
| 46 | 12 | 0 | 0 |
| 47 | 72 | 0 | 0 |
| 48 | 65 | 0 | 0 |
| 49 | 111 | 0 | 0 |
| 50 | 91 | 0 | 0 |
| 51 | 19 | 0 | 0 |
| 52 | 1 | 0 | 0 |
| 53 | 20 | 0 | 0 |
| 54 | 71 | 0 | 0 |
| 55 | 20 | 0 | 0 |
| 56 | 5 | 0 | 0 |
| 57 | 45 | 0 | 0 |
| 58 | 17 | 0 | 0 |
| 59 | 66 | 0 | 0 |
| 60 | 9 | 0 | 0 |
| 61 | 25 | 0 | 0 |
| 62 | 8 | 0 | 0 |
| 63 | 44 | 0 | 0 |
| 64 | 12 | 0 | 0 |
| 65 | 24 | 0 | 0 |
| 66 | 15 | 0 | 0 |
| 67 | 9 | 0 | 0 |
| 68 | 9 | 0 | 0 |
| 69 | 5 | 0 | 0 |
| 70 | 12 | 0 | 0 |
| 71 | 87 | 0 | 0 |
| 72 | 17 | 0 | 0 |
| 73 | 32 | 0 | 0 |
| 74 | 50 | 0 | 0 |
| 75 | 14 | 0 | 0 |
| 76 | 45 | 0 | 0 |
| 77 | 34 | 0 | 0 |
| 78 | 10 | 0 | 0 |
| 79 | 8 | 0 | 0 |
| 80 | 4 | 0 | 0 |
| 81 | 12 | 0 | 0 |
| 82 | 5 | 0 | 0 |
| 83 | 3 | 0 | 0 |
| 84 | 3 | 0 | 0 |
| 85 | 8 | 0 | 0 |
| 86 | 2 | 0 | 0 |
| 87 | 10 | 0 | 0 |
| 88 | 1 | 0 | 0 |
| 89 | 1 | 0 | 0 |
| 90 | 3 | 0 | 0 |
| 91 | 1 | 0 | 0 |
| 92 | 1 | 0 | 0 |
